# Transitioning to a national health system in Cyprus: a stakeholder analysis of pharmaceutical policy reform

**DOI:** 10.2471/BLT.14.148742

**Published:** 2015-06-18

**Authors:** Olivier J Wouters, Panos G Kanavos

**Affiliations:** a LSE Health and Social Care, London School of Economics and Political Science, Houghton Street, London WC2A 2AE, England.

## Abstract

**Objective:**

To review the pharmaceutical sector in Cyprus in terms of the availability and affordability of medicines and to explore pharmaceutical policy options for the national health system finance reform expected to be introduced in 2016.

**Methods:**

We conducted semi-structured interviews in April 2014 with senior representatives from seven key national organizations involved in pharmaceutical care. The captured data were coded and analysed using the predetermined themes of pricing, reimbursement, prescribing, dispensing and cost sharing. We also examined secondary data provided by the Cypriot Ministry of Health; these data included the prices and volumes of prescription medicines in 2013.

**Findings:**

We identified several key issues, including high medicine prices, underuse of generic medicines and high out-of-pocket drug spending. Most stakeholders recommended that the national government review existing pricing policies to ensure medicines within the forthcoming national health system are affordable and available, introduce a national reimbursement system and incentivize the prescribing and dispensing of generic medicines. There were disagreements over how to (i) allocate responsibilities to governmental agencies in the national health system, (ii) reconcile differences in opinion between stakeholders and (iii) raise awareness among patients, physicians and pharmacists about the benefits of greater generic drug use.

**Conclusion:**

In Cyprus, if the national health system is going to provide universal health coverage in a sustainable fashion, then the national government must address the current issues in the pharmaceutical sector. Importantly, the country will need to increase the market share of generic medicines to contain drug spending.

## Résumé

**Objectif:**

Examiner le secteur pharmaceutique à Chypre en termes de disponibilité et d'accessibilité économique des médicaments et étudier les options de politiques pharmaceutiques envisageables pour la réforme du financement du système de santé national, prévue pour 2016.

**Méthodes:**

Nous avons réalisé des entretiens semi-directifs en avril 2014 auprès de responsables représentant sept organisations nationales clés impliquées dans les soins pharmaceutiques. Les données obtenues ont été codées et analysées en utilisant les thèmes prédéfinis suivants: tarification, remboursement, prescription, délivrance et participation aux coûts. Nous avons également étudié des données secondaires fournies par le Ministère de la Santé chypriote; des données qui incluaient les prix et volumes des médicaments délivrés sur ordonnance en 2013.

**Résultats:**

Nous avons identifié plusieurs problèmes essentiels, notamment les prix élevés des médicaments, une sous-utilisation des médicaments génériques et des débours directs importants. La plupart des parties prenantes ont recommandé que le gouvernement national revoie les politiques de tarification existantes pour que les médicaments soient disponibles et économiquement accessibles dans le nouveau système de santé, qu'il mette en place un système de remboursement national et qu'il incite à prescrire et à délivrer des médicaments génériques. Des désaccords sont apparus sur la manière (i) d'affecter les responsabilités entre les différents organismes gouvernementaux dans le nouveau système de santé national, (ii) de réconcilier les divergences d'opinions des parties prenantes et (iii) de sensibiliser les patients, les médecins prescripteurs et les pharmaciens sur les avantages d'une plus grande utilisation des médicaments génériques.

**Conclusion:**

À Chypre, si l'on veut que le nouveau système national de santé garantisse une couverture sanitaire universelle de manière durable, il faudra d'abord que le gouvernement national résolve les problèmes qui existent actuellement dans le secteur pharmaceutique. Un élément déterminant consistera à augmenter la part de marché des médicaments génériques afin de contenir les dépenses en médicaments.

## Resumen

**Objetivo:**

Revisar el sector farmacéutico en Chipre en términos de la disponibilidad y asequibilidad de los medicamentos y explorar las opciones de la política farmacéutica para la reforma financiera del sistema nacional de salud que se prevé introducir en 2016.

**Métodos:**

En abril de 2014 se llevaron a cabo entrevistas semiestructuradas con los principales representantes de siete organizaciones nacionales clave involucradas en la atención farmacéutica. Se cifraron los datos obtenidos y se analizaron utilizando los temas predeterminados de fijación de precios, reembolso, prescripción, dispensación y reparto de costes. También se examinaron los datos secundarios proporcionados por el Ministerio de Salud de Chipre, los cuales incluían el precio y el volumen de los medicamentos recetados en 2013.

**Resultados:**

Se identificaron varias cuestiones clave, entre las que se incluyen el alto precio de los medicamentos, la poca utilización de medicamentos genéricos y el alto gasto en el desembolso de fármacos. La mayoría de los interesados recomendaron que el gobierno nacional revisara las políticas de fijación de precios existentes para asegurar que los medicamentos dentro del inminente sistema nacional de salud fuesen accesibles y asequibles, que introdujera un sistema de reembolso nacional y que incentivara la prescripción y distribución de medicamentos genéricos. Hubo desacuerdos en cuanto al modo de (i) asignar responsabilidades a las agencias gubernamentales en el sistema nacional de salud, (ii) resolver las diferencias de opinión entre los interesados y (iii) fomentar la sensibilización en cuanto a los beneficios de un mejor uso de los fármacos genéricos entre los pacientes, médicos y farmacéuticos.

**Conclusión:**

En Chipre, si el sistema nacional de salud quiere proporcionar cobertura sanitaria universal de un modo sostenible, el gobierno nacional tendrá que tratar los problemas actuales del sector farmacéutico. Otro aspecto importante es que el país necesitará aumentar la cuota de mercado de los medicamentos genéricos si quiere contener el gasto en fármacos.

## ملخص

**الغرض:**

مراجعة القطاع الدوائي في قبرص من حيث توفر الأدوية ومعقولية أسعارها، واستكشاف خيارات السياسة الدوائية المتاحة لإصلاح الجوانب المالية للنظام الصحي الوطني المزمع تنفيذه في عام 2016.

**الطريقة:**

أجرينا مقابلات شبه مقننة في أبريل/نيسان من عام 2014 مع كبار الممثلين من سبع مؤسسات وطنية بارزة تشارك في الرعاية الدوائية. وتم تشفير البيانات المسجّلة وتحليلها باستخدام الموضوعات المحددة سلفًا والمتعلقة بالتسعير ورد التكاليف ووصفات الأطباء وصرف الأدوية والمشاركة في التكلفة. كما نظرنا أيضًا في البيانات الثانوية التي قدمتها وزارة الصحة القبرصية، وقد تضمنت هذه البيانات أسعار الأدوية التي يصفها الأطباء وكمياتها في عام 2013.

**النتائج:**

لقد حددنا عدة مشكلات أساسية، بما يشمل الأسعار المرتفعة للدواء، وعدم الانتفاع الكافي بالأدوية المكافئة، وحجم الإنفاق المرتفع من جانب المرضى على الأدوية. وقد أوصت أغلب الجهات المعنية بأن تتولى الحكومة الوطنية مراجعة سياسات التسعير القائمة لضمان أن تكون الأدوية المقدمة في إطار النظام الصحي الوطني المُقبِل متوفرة وبتكلفة معقولة، وطرح نظام وطني لرد التكاليف، وتقديم الحوافز المشجعة على وصف الأدوية المكافئة وصرفها. وقد نشأت خلافات حول كيفية (أ) إسناد المسؤوليات للجهات الحكومية في النظام الصحي الوطني، و(ب) التوفيق بين الاختلافات في الآراء ما بين الجهات المعنية، و(جـ) رفع درجة الوعي بين المرضى والأطباء والصيادلة بشأن المنافع المرتبطة بزيادة الانتفاع بالأدوية المكافئة.

**الاستنتاج:**

إذا كان النظام الصحي الوطني في قبرص يعتزم تقديم تغطية صحية عامة بطريقة مستدامة، فعلى الحكومة الوطنية أن تتعامل مع المشكلات الحالية في القطاع الدوائي. والأهم هو أنه سيتعين على قبرص زيادة حصة السوق الممنوحة للأدوية المكافئة لتشمل الإنفاق على الأدوية.

## 摘要

**目的:**

旨在评审塞浦路斯境内医药部门的药品可用性和可负担性，并且针对有望在 2016 年引进的国家卫生系统财政改革探讨医药政策选择。

**方法:**

我们与参与医药保健的七个主要国家组织的高级代表一起在 2014 年 4 月开展了一项半结构性访谈。 通过采用定价、报销、开处方、配药和成本分担这些预先设定的主题，我们对所获取的数据进行了编码和分析。 我们还研究了由塞浦路斯卫生部提供的次级数据；在 2013 年的处方药价格及销量中包含这些数据。

**结果:**

我们确定了几个关键问题，包括药品价格高、非专利药品未得到充分利用，以及自费药品支出高。 大多数利益相关者建议国家政府应评审现有价格政策，以确保药品在即将设立的国家卫生系统中具有可负担性和可用性，同时应引进国家报销系统，并且采取奖励措施以鼓励在开方和配药时采用非专利药品。 以下几点存在分歧：(i) 如何分配政府机构在国家卫生系统中承担的责任，(ii) 如何使利益相关者之间的看法差异达成一致，以及 (iii) 如何使患者、医师和药剂师在更加广泛地使用非专利药品方面提高意识。

**结论:**

在塞浦路斯，如果国家卫生系统要以可持续发展的方式提供全民医疗保险，那么国家政府就必须解决医药部门目前出现的问题。 重要的是，该国将需提高非专利药品的市场份额，以遏制药品支出。

## Резюме

**Цель:**

Рассмотреть состояние фармацевтического сектора на Кипре с точки зрения доступности медикаментов по ассортименту и цене и изучить возможности фармацевтической политики в плане реформы финансирования национальной системы здравоохранения, которая, как ожидается, будет проведена в 2016 г.

**Методы:**

В апреле 2014 г. были проведены частично структурированные опросы представителей высшего руководства семи основных национальных организаций, вовлеченных в оказание лекарственной помощи населению. Собранные данные были переведены в цифровой формат и проанализированы с точки зрения заранее определенных аспектов: ценообразования, возмещения, выдачи рецептов, отпуска и распределения затрат. Мы также изучили вторичные данные, предоставленные Министерством здравоохранения Кипра, которые включали сведения о ценах и количестве рецептурных препаратов, выписанных в 2013 г.

**Результаты:**

Были выявлены несколько основных проблем, включая высокие цены на лекарства, недостаточное использование дженериков и слишком частое использование собственных средств на оплату лекарств. Большинство партнеров предложили национальному правительству пересмотреть существующую политику ценообразования, чтобы в будущей системе здравоохранения лекарства были более доступны по цене и ассортименту, ввести национальную систему возмещения затрат на медикаменты и поощрять назначение и отпуск дженериков. Имелись разногласия по поводу того, как (i) распределить ответственность между правительственными органами в национальной системе здравоохранения, (ii) примирить разногласия во мнениях между партнерами и (iii) предоставить пациентам, врачам и фармацевтам больше информации о пользе дженериков.

**Вывод:**

Если национальная система здравоохранения на Кипре намерена стабильно предоставлять населению общедоступные услуги по охране здоровья, национальному правительству следует обратить внимание на текущие проблемы фармацевтического сектора. Важно, что страна должна будет увеличить рыночную долю дженериков, чтобы уменьшить затраты на медицинские препараты.

## Introduction

In 2013, Cyprus had a population of about 858 000 and a gross domestic product (GDP) of about 16 500 euros (€) per capita.^1^^,^^2^ The country's health system consists of a public and a private sector. Individuals with annual incomes of no more than €15 400, the chronically ill and civil servants – together representing about 83% of the population – are eligible for public-sector coverage.^3^ The government pays for public-sector health care while patients and private health insurers pay for private-sector health care. Total health expenditure is about 7.3% of GDP.^4^ About 43% and 57% of health spending is publicly and privately funded, respectively.^1^ In 2010, pharmaceutical expenditure – €322 per capita – accounted for 19.8% of total health expenditure in Cyprus.^1^

In 2013, Cyprus agreed to a memorandum of understanding with creditors from the European Commission, European Central Bank and International Monetary Fund and introduced an economic adjustment programme to address the country’s financial, fiscal and structural challenges.^5^ The memorandum calls for the introduction of a national health system finance reform by mid-2016 to allow free choice of provider, social equality and solidarity, financial sustainability, and universal coverage of a minimum benefit basket.^6^ In the forthcoming system, the government will pay for all health-care services in the benefit basket – subject to cost sharing – and supplement current tax revenues with other sources of funding, including taxes on employers, employees and pensioners.^7^ The reform will bring major changes in financing, coverage, provider payment and data collection and monitoring.^3^ The government still needs to decide which drugs to cover, which pricing and reimbursement policies to apply and what type of cost sharing to introduce.

Given the lack of research on the Cypriot pharmaceutical system,^8^^–^^12^ the aim of this study was to review the current system of pharmaceutical care in the private and public sectors in terms of the availability and affordability of medicines. We also wanted to explore how the public and private markets could be efficiently merged in the national health system and to assess the key barriers to the implementation of the new system.

## Methods

To collect primary data, we conducted interviews in April 2014 with senior representatives from seven national organizations (Box 1).^13^ The interviewees represented all but one of the organizations involved in pharmaceutical care in Cyprus. The exception was the Cyprus Medical Association, whose representatives were unavailable to meet. The interviewees were jointly selected by the researchers, the World Health Organization Regional Office for Europe and the Cypriot Ministry of Health. We met with the representatives from each organization separately over three days and each interview lasted between 30 minutes and two hours. All interviews were held at the headquarters of the health ministry's Department of Pharmaceutical Services, in Nicosia. At least three members of this department were present at each interview.

Box 1 **National organizations represented by interviewees, Cyprus, 2014**

- Cyprus Association of Pharmaceutical Companies, representing Cypriot drug importers and distributors.
- Cyprus Association of Research and Development Pharmaceutical Companies, representing research-based manufacturers.
- Cyprus Pharmaceutical and Chemical Manufacturing Company, representing Cypriot manufacturers of generic drugs.
- Cyprus Pharmaceutical Manufacturer Association, representing Cypriot pharmacists.
- The Health Insurance Organization, the government agency in charge of implementing the national health system reforms.
- Pancyprian Federation of Patients Associations and Friends, representing Cypriot patients.
- Ministry of health's Department of Pharmaceutical Services, the government department in charge of national pharmaceutical policies.

The interviews were semi-structured (Box 2) but the discussions varied based on the roles of each organization. One of the researchers and a ministry of health employee took notes during each interview, and these notes were discussed with health ministry officials after each meeting, to confirm our understanding of the data. We followed the consolidated criteria for reporting qualitative research checklist^14^ and used NVivo 10 (QSR International, Melbourne, Australia) to organize, code and analyse the interview data.

Box 2 **Semi-structured interview template used to assess the Cypriot pharmaceutical market, Cyprus, 2014**

- What are the strengths and weaknesses of the pharmaceutical policies in the public sector?
- What are the strengths and weaknesses of the pharmaceutical policies in the private sector?
- Which pharmaceutical policies should be changed before the introduction of the national health system reforms?
- Which pharmaceutical policies should be applied in the national health system?
- What are the key barriers to the successful implementation of the national health system reforms?

The Department of Pharmaceutical Services also provided secondary data to help us understand the current policies and features of the pharmaceutical markets. These data included the prices and volumes of all prescription medicines used in the public and private sectors in 2013, relevant legislative documents and internal ministry of health reports. The quantitative data were analysed using Excel 2007 (Microsoft, Redmond, United States of America).

## Results

### Current pharmaceutical policies

#### Public sector

Public-sector drugs, which are freely available to patients with public health insurance, are procured centrally by the ministry of health through two types of tenders: open invitations and negotiations.^10^^,^^11^

In an open invitation, which is used for about 75% of the drugs consumed in the public sector, the ministry of health issues a request for a quantity of drugs and invites confidential bids from manufacturers worldwide. The manufacturer that offers the lowest price is then asked to supply the entire market for two years. A tender category usually includes a single molecule – i.e. the originator brand drug and generic drugs with the same active ingredient – but may also include all drugs that treat the same condition – e.g. the class of cholesterol-reducing drugs known as statins. The invitation process lasts about eight months – excluding drug delivery time – and accounted for €54.5 million of government expenditure in 2013. The remaining 25% of drugs used in the public sector, which are mostly on-patent, are procured through negotiations and accounted for €50 million of government expenditure in 2013. Once a tender price has been accepted by both the ministry of health and the manufacturer, it is legally binding and cannot be changed.

The public-sector tender prices of generic drugs are usually 20–70% lower than the private-sector wholesale prices. In extreme cases, prices in the private sector may be more than 30-fold higher than in the public sector (Table 1). For on-patent drugs, however, the public-sector prices are usually only 5–10% lower than the private-sector prices.

**Table 1:** Anonymized tender results for three selected medicines, Cyprus, 2013

Product, condition, bid	Quantity, packs	Bid price, €/pack	Budget impact, €^a^	Private-sector wholesale price, €/pack
**Product A (hypertension)**				
Bid 1 (winner)	36 000 000	0.0189	678 857	0.35
Bid 2		0.0223	804 000	
Bid 3		0.0225	801 000	
Bid 4		0.0239	861 428	
Bid 5		0.0260	936 000	
Bid 6		0.0333	1 200 000	
Bid 7		0.0411	1 478 571	
Bid 8		0.0463	1 668 215	
Bid 9		0.1500	5 399 999	
**Product B (osteoporosis and other bone disease)**				
Bid 1 (winner)	5 000	12.00	60 000	208.02
Bid 2		29.29	146 450	
Bid 3		29.50	147 500	
Bid 4		29.85	149 250	
Bid 5		33.97	169 850	
Bid 6		38.90	194 500	
Bid 7		50.09	250 439	
Bid 8		105.00	525 000	
Bid 9		129.00	645 000	
**Product C (colorectal cancer)**				
Bid 1 (winner)	2 800	9.12	25 536	50.00
Bid 2		12.00	33 600	

€: euros.

^a^ Actual budget impact may vary due to rounding.

Source: Data provided by the Department of Pharmaceutical Services, Ministry of Health, Nicosia, Cyprus.

For all tenders, the government buys the stock in three to four instalments and distributes the drugs to the 11 hospital and 34 retail pharmacies in Cyprus, which together represent one public pharmacy for every 15 500 public-sector patients. Public-sector pharmacists receive a government salary. The annual storage, distribution and dispensing costs for drugs sold in retail pharmacies total about €6.3 million.

Table 2 summarizes the drug expenditure in the public sector for the year 2013; the 10 and 50 highest-selling products accounted for 17.6% and 44.0% of expenditure, respectively. In the same year, there were 18 foreign research-based manufacturers that each had over €1 million in public-sector drug sales in Cyprus – together representing 56.0% of all such sales. All foreign manufacturers sell their drugs in Cyprus via about 45 importers. These importers serve as wholesalers and handle national pharmacovigilance requirements. There are three Cypriot generic drug manufacturers, which export as much as 93% of their output to foreign markets.

**Table 2:** Drug expenditure in the public and private sectors, Cyprus, 2013

Category	Expenditure (millions of euros)^a^	
	Public sector	Private sector
**Prescription drugs**^b^	98.5	80.6
Inpatient	59.5	9.7
Outpatient	39.0	70.9
On-patent originator brand	7.7	8.4
Off-patent originator brand	10.8	46.6
Generic	19.3	11.4
Vaccines and others	1.2	4.5
**Over-the-counter drugs**	5.0	14.3
**Total drug expenditure**	**103.5**	**94.9**

^a^ Excluding value added tax.

^b^ Inpatient and outpatient drugs are sold in hospital and retail pharmacies, respectively.

Source: Data provided by the Department of Pharmaceutical Services, Ministry of Health, Nicosia, Cyprus.

All drugs sold in the Cypriot public sector are listed in a national formulary, which included 1767 products in 2013. Nearly all of the drugs used in Cyprus for the treatment of cancer, haemophilia, hepatitis B, hepatitis C and human immunodeficiency virus are sold exclusively in public pharmacies because all patients with these illnesses are eligible for public coverage. There is a co-payment plan, with an annual budget of €600 000, that allows public-sector patients to buy medicines only available in the private sector.

#### Private sector

Private-sector drug prices are set by the health ministry based on the recommendations of a pricing committee. For on-patent products, this committee bases the Cypriot wholesale price on the mean of the wholesale prices in one high-price country – i.e. Sweden, two medium-price countries – i.e. Austria and France, and one low-price country – i.e. Greece. If a medicine is not available in one of these countries, the committee uses the price in a pre-selected alternate country. To account for the cost of importing the drug into Cyprus, the committee adds a 3% mark-up to the derived mean price. The committee recalculates the prices of most drugs every two years. It revises the price of each newly launched product annually for the first two years. The private-sector prices in Cyprus are among the highest in Europe,^15^ largely because this pricing system captures the official prices in the reference countries and does not take into account confidential discounts.

After patent expiry, originator brand drugs continue to be priced through international price referencing. Generic drugs must be priced at least 20% below the price of the originator brand at the time of patent expiry. Consumption of generic drugs in the private sector is low, partly because pharmacists are forbidden by law to substitute such drugs for any originator brand drugs prescribed by physicians (Box 3).

Box 3 **Current issues in the Cypriot pharmaceutical market**

- The private-sector prices are among the highest in Europe, largely because international price referencing does not capture confidential discounts in other countries.
- On-patent drugs in the public sector are expensive. As public-sector prices are published online – and may therefore influence prices in countries that use the Cypriot prices for reference – manufacturers are not willing to provide large discounts to the ministry of health. The national association for research-based manufacturers has confirmed this observation.
- There is underuse of generic drugs in the private sector and generic substitution by pharmacists is forbidden. Over 77% of spending in private retail pharmacies is on branded products – i.e. on-patent and off-patent originator brands.
- Although there is a national list of approved pharmaceutical products, the government does not disseminate any prescribing guidelines and there are no information systems to monitor or control prescribing behaviour.
- There are few limits on the financial relationships between physicians and manufacturers, which may lead to conflicts of interest.
- Private-sector patients pay for drugs almost entirely out-of-pocket. This could expose patients to undue financial risks or deter some from seeking beneficial treatment.

In 2013 there were 481 private pharmacies in Cyprus – i.e. about one for every 300 private-sector patients. The pharmacy price of a drug includes the pharmacist’s mark-up and a value added tax of 5%. The mark-up is determined by the wholesale price of the drug pack and is set at 37%, 33% and 25% for packs that cost no more than €50, between €50 and €250, and more than €250, respectively. Private-sector pharmacists also charge a flat fee of €1.00 per prescription.

Table 2 summarizes the 2013 drug expenditure in the private sector. The 10 and 50 highest-selling products accounted for 11.5% and 34.5% of private drug spending, respectively. About 87% of the total health expenditure within the Cypriot private sector was out-of-pocket while private health insurers paid the rest.^4^ Only 2054 of the 5241 products registered for sale in the private sector were available in 2013 – mostly because of insufficient demand for the other products.

### Policy options

We investigated pharmaceutical policy options for the national health system, dividing the main feedback and suggestions of the stakeholders into the categories of pricing, reimbursement, prescribing, dispensing and cost sharing. Below, to contextualize the stakeholders’ statements, we have added references to relevant studies.

#### Pricing

The consensus was that reviewing the current pricing policies to facilitate the transition to the national health system was important. To decrease the prices of on-patent drugs in the public sector, the association representing research-based manufacturers recommended the ministry of health keep price discounts confidential – thus limiting the spill-over effect on markets that use Cypriot prices for reference. The health ministry representatives agreed to investigate legal options that could be followed to strike confidential agreements on drug prices. To reduce private-sector prices, the ministry of health offered to adjust its system of international price referencing – e.g. it could apply the lowest price paid in the reference countries.

Stakeholders held differing views about which pricing policy to follow. The national associations for drug importers, local generic drug manufacturers, pharmacists and research-based manufacturers each noted that there is a possible trade-off between low prices and the availability of medicines. As Cyprus is a small market, these groups posited that, if prices drop too low, the manufacturers of originator brand and generic drugs might not sell their products in Cyprus – because it would produce insufficient returns on the manufacturers’ investments and/or adversely affect prices in other markets that use Cypriot prices for reference. The same groups urged the Cypriot government to use international price referencing in any future national health system and to apply a reimbursement system to receive confidential discounts. Other things being equal, however, a small population size does not appear to be associated with a relatively low market penetration by generic drugs.^16^

The ministry of health claimed the current wholesale prices of drugs in the private sector would be unaffordable in a national health system and that tendering could be used more widely. The Health Insurance Organization suggested the prices of drugs in the national health system should be set somewhere between the current public- and private-sector prices, but did not elaborate further.

#### Reimbursement

All stakeholders were in favour of introducing a national reimbursement system. The Health Insurance Organization intends to create a new national formulary and a reimbursement committee to manage it. Formularies can be used to specify the medicines eligible for reimbursement and – alongside prescribing guidelines – encourage the rational use of medicines.^17^

The Health Insurance Organization and ministry of health are working independently on criteria for the admission of new products to a future formulary. The ministry of health suggested that, to guide the inclusion or non-inclusion of drugs in a national formulary, the government should monitor, collect and analyse all relevant clinical and economic evidence from health technology assessment bodies in other countries. The government could ask manufacturers to adapt foreign data on the cost–effectiveness of drugs to local conditions.

The association for research-based manufacturers favoured the use of risk-sharing schemes in the national health system. Such schemes could be applied to hedge against uncertainties – at the time of a drug’s entry to the Cypriot market – regarding the drug’s budget impact, clinical effectiveness and cost–effectiveness. These schemes grant manufacturers favourable reimbursement rates in return for achieving financial or outcome targets. The Health Insurance Organization is considering the use of risk-sharing schemes. Although such schemes are widely used in Europe, they require appropriate performance measurement and enforcement.^18^^,^^19^

Finally, the Health Insurance Organization noted that widespread tendering in a unified national market could create supply disruptions, drive some generic drug manufacturers out of business and lead to higher generic drug prices over time. The organization proposed instead to use internal reference pricing and to tender selectively if such pricing does not achieve adequate price reductions for some products. Internal reference pricing sets a reimbursement ceiling based on the prices in a basket of drugs – e.g. the mean price of all drugs with the same active ingredient. If the price of a drug exceeds the reference price, the patient usually has to pay the difference. Systematic reviews have consistently found that such a policy can reduce drug prices and generate savings.^20^^–^^22^ The federation representing patients supported offering patients the choice between a generic drug and an originator brand version at a higher price.

#### Prescribing

Prescribing guidelines can have a beneficial impact on prescribing, when enforced appropriately.^23^^,^^24^ The Cypriot Ministry of Health plans to develop such guidelines for conditions with a high budget impact. When appropriate, the ministry might adapt guidelines published in other countries.

The interviewed representatives of the ministry of health, the Health Insurance Organization and pharmacy association suggested the government enforce the prescribing of generic drugs in the national health system. The Health Insurance Organization aims to introduce an electronic prescribing system to examine prescribing patterns and to improve the quality of medicine use. The organization is reviewing other options to encourage cost–effective prescribing, such as pay-for-performance schemes. It remains unclear, from the evidence collected in other countries, whether pay-for-performance schemes often achieve their intended goals.^25^

The Cypriot Ministry of Health believes there should be appropriate limits on drug advertising and on the gifts and contributions given to physicians by drug manufacturers. One survey has found that, for Cypriot physicians, pharmaceutical sales representatives are one of the most important sources of information on the safety and efficacy of medicines.^26^

#### Dispensing

In some countries, if a physician prescribes an originator brand drug despite the availability of a cheaper generic equivalent, pharmacists can override the physician’s decision and dispense the generic drug instead. Depending on the country, such generic substitution can be mandatory,^27^ voluntary^28^ or, as in Cyprus, forbidden. In our interviews, both the ministry of health and the Health Insurance Organization favoured mandatory generic substitution, which can speed up the market entry of generic drugs and reduce pharmaceutical spending.^29^ The federation representing patients opposed such substitution, however, and stated that all treatment decisions should be made by physicians.

Most (81.3%) sales in the Cypriot private sector in 2013 were for drug packs with a wholesale price of no more than €50 per pack – these packs were subject to one of the highest pharmacy mark-ups in Europe, of 37%.^30^ The ministry of health and the Health Insurance Organization stressed that pharmacy mark-ups needed to be reduced and revised in Cyprus to encourage the dispensing of generic drugs. However, the interviewees from the association representing pharmacists expressed concern about the poor macroeconomic conditions in Cyprus and, consequently, the financial viability of pharmacies if the remuneration system were to change in any way that would reduce the income of pharmacists.

#### Cost sharing

The Health Insurance Organization is exploring various cost-sharing options – i.e. deductibles, co-insurance or co-payments or any combination of these. The organization is also considering whether to apply exemption criteria and cost-sharing caps to protect patients financially. It may remove co-payments for conditions where compliance is an issue, such as some psychiatric conditions. The interviewees from the federation representing patients stressed the importance of limits on cost sharing to protect vulnerable groups like the chronically ill. The interviewees from the ministry of health stated that the current out-of-pocket burden on private-sector patients was too high and that this burden needed to be reduced in the national health system.

### Barriers

We identified four key barriers to the successful implementation of a comprehensive drug-benefit plan in the forthcoming national health system reforms.

First, it appeared difficult to obtain the buy-in of all stakeholders for the health-care reform. Notably, there was disagreement over whether the prices of prescription medicines in the future system should be the current private-sector or public-sector prices or lie somewhere between the two. Other disputes might arise, such as physicians resisting the monitoring of prescribing habits. To resolve such disputes, it is important to involve all stakeholders in the reform process.

Second, the governmental stakeholders – i.e. the Health Insurance Organization and ministry of health – need to clarify their roles in the forthcoming system, particularly regarding who will be in charge of reimbursement. Clear and transparent rules are needed to allocate responsibilities. Since its inception, the Cypriot Ministry of Health has been solely in charge of national pharmaceutical policies. Although the Health Insurance Organization was established in 2001,^7^ it has only been actively engaged in discussions with the ministry of health for the last few years.

Third, most of the proposed policy changes would need to be accompanied by legislative changes, which may be time-consuming. Although the memorandum of understanding provided a broad timeline for the implementation of a national health system – including deadlines for key legislative changes – it allowed little time for consensus-building and preparation.

Finally, the Pancyprian Federation of Patients Associations and Friends stated that many patients – especially in the private sector – do not perceive generic drugs to be as good as the originator brand drugs in terms of safety and efficacy. It is possible that in Cyprus some physicians and pharmacists also exhibit loyalty to originator brand medicines. Such perceptions and brand loyalty have been observed elsewhere^31^^,^^32^ and may explain why generic substitution has been forbidden in the Cypriot private sector. The government could launch a public education campaign to promote the use of generic drugs.^33^

## Discussion

Pharmaceutical policies should reflect national priorities for health and industrial policy, including cost containment, employment, innovation and trade promotion.^34^ In many countries, the main objectives of pharmaceutical policies are to ensure equitable access to – and the good quality and rational use of – effective drugs.^35^ The findings of this study are meant to inform the ongoing policy deliberations in Cyprus. They can also be used to inform discussions in other countries aiming to establish a comprehensive drug-benefit plan under universal health coverage.

This study has some limitations. First, personal bias is unavoidable in interviews. To minimize the risk of such bias, both interviewers closely followed an interview template. Second, no representatives of the Cyprus Medical Association were available for an interview during the study visit. Members of this association could have provided valuable input on the prescribing environment. Finally, although this study looked at reform in the pharmaceutical sector, a holistic analysis is needed to understand the full impact of national health system reforms in Cyprus.

Over the next few years, there is a need to update the legislative and institutional framework in Cyprus and to acquire data, through pilot studies and simulations, on how health care might operate under the new system. There is a further need to build capacity and to address issues before and after reforms are introduced. The government should work to eliminate each of the four barriers identified. The Cypriot authorities should also prepare for unforeseen problems that inevitably accompany large-scale changes to health systems. Once new policies are implemented, the government should continue to monitor the results.
